# Retraction: Long non-coding RNA SNHG14 exerts oncogenic functions in non-small cell lung cancer through acting as a miR-340 sponge

**DOI:** 10.1042/BSR-2018-0941_RET

**Published:** 2024-04-29

**Keywords:** long non-coding RNA, miR-30, non-small cell lung cancer, SNHG14

This article is being retracted from *Bioscience Reports* at the request of the Editor-in-Chief and the Editorial Board following receipt of a notification from a reader, alerting the Editorial Board to multiple similarities between figures in this paper and papers by different authors. Specifically:

- Figure 2D si-NC panel similarities with Figure 3A SGC-7901 0 panel from Hu et al, 2019 (DOI: 10.1002/2211-5463.12693) [retracted], Figure 4A HCT-116 RP11-708H21.4 panel from Sun et al, 2017 (DOI: 10.18632/oncotarget.15846), Figure 5D E2+AZD9496 panel and Figure 6E NC panel from Liu et al, 2020 (DOI: 10.1042/BSR20191330) [retracted]
- Figure 4B similarities with Figure 6 OE-HOXA10+sh-STAT3 panel from Chen et al, 2019 (DOI: 10.2147/cmar.s201342), Figure 6 NSP A2780 from Ruan et al, 2019 (DOI: 10.2147/cmar.s180418) [retracted], Figure 6 OE-LIN28B+si-BCL-2 and OE-LIN28B+si-NC panels from Yuan et al, 2018 (DOI: 10.1016/j.biopha.2018.04.002), Figure 8C from Wang et al, 2019 (DOI: 10.2147/cmar.s177508) [retracted], and Figure 2B from Hu et al, 2019 (DOI: 10.1002/2211-5463.12693) [retracted]

The authors have been contacted with regards to the retraction and have not responded to the Journal's queries or the concerns raised. Given the extent of the issues raised, the Editorial Board stand by the decision to retract the article.

